# Persistence of Intimate Partner Violence and Its Relationship to HIV Prevention Behaviors and Attitudes: A Longitudinal Study of Coupled Sexual Minority Men

**DOI:** 10.1007/s10508-025-03363-4

**Published:** 2026-02-25

**Authors:** Erik D. Storholm, Jessica L. Randazzo, Daniel Siconolfi, Glenn J. Wagner

**Affiliations:** 1 School of Public Health, San Diego State University, San Diego, CA USA; 2 Health, RAND Corporation, 1776 Main Street, Santa Monica, CA 90407 USA

**Keywords:** IPV, PrEP, Stigma, Non-monogamy, HIV testing, Sexual orientation

## Abstract

Attitudes toward and use of pre-exposure prophylaxis (PrEP) by coupled sexual minority men (SMM) may be influenced by exposure to intimate partner violence (IPV) and sexual agreements regarding non-monogamy. We examined IPV (victimization and perpetration) persistence over 12 months and its association with HIV prevention behaviors and attitudes among 271 coupled, HIV-negative SMM, and the moderating role of sexual agreements. The sample had diverse race/ethnicity (64% were non-White) and was from across all regions of the USA. Participants completed a self-administered, Web-based survey at baseline, 6 months, and 12 months, and reported having the same main partner throughout. Repeated measures regression analysis showed that the level of IPV persistence was unrelated to PrEP use; however, persistent (i.e., present at all three assessments) IPV victimization and perpetration both predicted lower perceived partner support for PrEP use among those not using PrEP, as well as greater PrEP stigma. Persistent IPV victimization and perpetration were both predictive of more frequent HIV testing. The presence of a sexual agreement for non-monogamy (i.e., sex outside the primary relationship), which was a significant independent predictor of more HIV testing and PrEP use, moderated the relationships between IPV and HIV testing and partner support for PrEP, albeit in mixed ways: In non-monogamous relationships, persistent IPV victimization and perpetration were both associated with less HIV testing, but greater perceived partner support for PrEP use. In one of the first longitudinal studies of IPV and HIV prevention among SMM, these findings suggest a complex and nuanced influence of IPV on HIV prevention behaviors and attitudes and the key role that open communication and agreements related to extradyadic sex partners may have for HIV prevention among coupled SMM.

## Introduction

Sexual minority men (SMM) in the USA have long been disproportionately impacted by the HIV epidemic (Centers for Disease Control and Prevention [CDC]). Among the many factors associated with heightened HIV risk among SMM in the USA is intimate partner violence (IPV) (Braksmajer et al., 2020; Buller et al., 2014; Parsons et al., 2017; Storholm et al., 2023). Studies have shown that IPV is equally present among SMM as it is among heterosexual relationships, with roughly one-third of SMM reporting lifetime exposure to IPV victimization, and one in five reporting ever perpetrating IPV (Hong, 2024; Liu et al., 2021; Trombetta & Rollè, 2023; Walters et al., 2013).

Pre-exposure prophylaxis (PrEP) is perhaps the leading tool for HIV prevention in the USA, and it provides SMM who are experiencing IPV victimization with a prevention option that does not require condom negotiation with their partner or compromising safety. However, IPV may impede utilization of and access to health services such as PrEP and HIV testing through fear of stigma, discrimination, and shame associated with IPV disclosure (Stephenson et al., 2021), as well as IPV being associated with greater stigmatization of PrEP use (Braksmajer et al., 2020; Stephenson et al., 2021). A systematic review and meta-analysis published in 2024 of 13 studies examining the association between IPV and PrEP use among SMM, most of which were conducted in the USA, revealed mixed findings (Hong, 2024). Some studies showed that IPV was negatively associated with PrEP use and willingness to use PrEP (Hong et al., 2022, 2023; Wirtz et al., 2022), while other studies did not find significant associations (Blashill et al., 2020; Chen et al., 2021; Friedman et al., 2019). All the reviewed quantitative research was cross-sectional, and all but one study measured IPV victimization but not perpetration. The authors called for further research, including longitudinal studies, to better understand the complex relationship between IPV and PrEP use and its underlying mechanisms. Longitudinal studies of IPV will enable an examination of the role of persistent versus intermittent IPV over time and its association with HIV prevention behaviors.

Few studies have examined how relationship factors influence the association between IPV and PrEP-related attitudes, beliefs, and behaviors among coupled SMM. PrEP use among men in relationships may be influenced by partner support for PrEP, perceived benefits of PrEP for the relationship, and stigma related to PrEP use—all of which may be influenced by exposure to IPV (victimization and perpetration), as well as relationship factors such as sexual agreements regarding sex outside the relationship (Hong, 2024; Pico-Espinosa et al., 2022; Turpin et al., 2025). In a large sample of 659 coupled SMM, perceived partner support for the respondent using PrEP was negatively correlated with IPV victimization, but not significantly associated with IPV perpetration, after controlling for the presence of sexual agreements (Kahle et al., 2020). Furthermore, those who had an agreement with their partner that allowed sex outside the relationship were more likely to report partner attitudinal support for PrEP, and to perceive PrEP use as beneficial, after controlling for IPV victimization and perpetration. In relationships that do not allow extradyadic sexual partners, PrEP use may be stigmatized and interpreted by a partner to be an indication of infidelity and/or engagement in sexual risk behavior, which could lower trust and trigger violence and abuse (Kahle et al., 2020; Nguyen et al., 2021; Storholm et al., 2023).

We recently completed an online survey of 500 SMM residing across the USA who enrolled in project Empowering Relationships and Opportunities for Safety (EROS)—one of the first longitudinal studies of IPV and HIV prevention behaviors among SMM (Storholm et al., 2022). This paper reports analyses examining IPV (victimization and perpetration) persistence over 12 months, and its association with HIV prevention behaviors and attitudes among coupled SMM, and the moderating role of sexual agreements.

## Method

### Study Design

EROS is a longitudinal, observational study that examines how aspects of IPV and the psychosocial context surrounding IPV relate to the use of HIV prevention behaviors among SMM. The study follows enrolled men over 24 months, with assessments every six months. The study is still ongoing and will not be completed until mid-2026; therefore, the analysis reported here was conducted solely with data through month 12. Details of the study protocol are available elsewhere (Storholm et al., 2022).

### Participants

Participants enrolled in the study between September 2022 and December 2023. Recruitment was primarily (95% of participants) through online advertisements placed on social networking sites (i.e., Facebook and Instagram) and mobile dating/hookup apps (e.g., Scruff, Jack’d, Grindr), although flyers were also distributed at bars, gyms, pride events, and LGBTQ+ service organizations in several cities around the country. Potential participants who clicked on the online advertisements or who scanned the quick response (QR) code displayed on the physical flyers were directed to a short Qualtrics survey that provided information about the study, consent to be screened, and screening questions to assess eligibility.

We sought to recruit men who had heightened risk for exposure to IPV and HIV. Accordingly, to be eligible, prospective participants needed to report: (1) being cisgender male (assigned male sex at birth and currently identified as a man); (2) being in a relationship with another cisgender man for a minimum of three months at the time of screening (but not necessarily at the time of the baseline survey); (3) being 18–45 years of age; (4) having HIV-negative or unknown HIV status; (5) living in one of the Center for Disease Control and Prevention (CDC)-defined USA. Ending the HIV Epidemic (EHE) priority jurisdictions (Fauci et al., 2019); and (6) willingness to receive home-based HIV/STI test kits at a chosen address. To reduce the risk of violence resulting from participation in the study, participants were informed that only one partner in a relationship dyad was eligible to participate. Partner non-participation was verified prior to enrollment by collecting the partner’s first name and last 4 digits of their phone number from potential participants and checking these data against all enrolled participants. Verification of identity (using software, manual checks, and identity documentation) at multiple stages of the enrollment process was used to prevent identity bots, duplicate respondents, and fraudulent respondents.

After completing the initial screening, participants were not considered to be fully eligible until they completed the following three steps: (1) attended a live 15–20-min virtual onboarding session with a study coordinator, which consisted of identity verification (via identification card), orientation to the study, and obtaining written consent via electronic signature; (2) completed a 45–60 min online, self-administered baseline survey; and (3) returned a viable HIV/STI test kit to confirm HIV-negative status at baseline. Participants were remunerated $50 (e-gift certificates) for completion of all baseline tasks (virtual onboarding, virtual survey, returned home-based HIV/STI test kit). In total, 9010 unique persons responded to the study ad by completing a screener, 1042 were eligible for the study based on their screener data, 742 attended the virtual onboarding session, 690 completed the baseline survey, and 522 returned a viable test kit; 22 were not eligible due to an HIV seropositive test result; these participants were notified and immediately linked to HIV care. Therefore, a final sample of 500 SMM enrolled in the EROS cohort.

### Measures

The survey instrument was self-administered with a Web-based survey instrument. All measures were assessed at each assessment, unless noted otherwise.

*Intimate partner violence (IPV)*. We measured IPV with 62 items adapted from validated measures of IPV (Postmus et al., 2016; Stephenson & Finneran, 2013). For each item, respondents were asked to respond yes/no to whether they had experienced the behavior in the prior six months with respect to their current partner; each item was asked twice, to assess both victimization (if they experienced the abusive behavior from their partner) and perpetration (if they did the behavior to their partner). Unlike IPV measures used in prior research with SMM, we asked participants to exclude any behaviors that were part of consensual sexual behavior (e.g., role play), or that were done by accident. The behaviors span across eight domains of IPV: controlling (13 items; e.g., demanded access to phone, controlled access to family), emotional (7 items; e.g., criticized appearance, told you no one else would want you), financial (3 items; e.g., racked up debt in your name to intentionally damage your credit), identity-based (5 items; e.g., threatened to "out" you, call immigration services), physical (8 items; e.g., slapped, threw objects), sexual (6 items; e.g., raped, forced to watch pornography), sexual health (12 items; e.g., prevented from taking pre-exposure prophylaxis [PrEP], pressured to take off condom), and stalking (8 items; e.g., showed up to places unwanted, used technology to monitor). Immediately following this section of the survey, participants were provided with contact information for the Domestic Violence Hotline. At the end of the survey, all participants received a resource guide with national- and state-level resources for intimate partner violence, mental health, substance use, and sexual health. Dichotomous variables were created to indicate whether any IPV victimization and any IPV perpetration were reported, in separate variables.

*PrEP-related measures*. PrEP use was assessed by asking participants to indicate whether they had ever been on PrEP, and if so, whether they were currently on PrEP. Among those who had never used PrEP or were not currently on PrEP, we assessed perceived partner support for PrEP use by the respondent. This construct was assessed with an 11-item measure created in a previous survey assessing comfort discussing PrEP with partners and partner support for PrEP (Stephenson et al., 2022). Each item is a statement about the partner’s attitude toward or support for the respondent taking PrEP (e.g., "My partner would support me taking PrEP"; “My partner would react violently if I started taking PrEP”) and the respondent rated their agreement on a scale of 1 “strongly disagree” to 5 “strongly agree.” After reverse coding appropriately, a mean item score was calculated and higher scores reflect greater partner support; Cronbach’s alpha = 0.89. PrEP stigma was measured at baseline and month 12 with the 13-item HIV PrEP Stigma Scale (HPSS) (Siegler et al., 2020). This scale uses statements to assess the respondent’s stigma related to themselves and others using PrEP (e.g., “I would feel ashamed to take PrEP in front of others”; “People experience negative judgment because they take PrEP”); the respondent rates their agreement with each statement from 1 “strongly disagree” to 5 “strongly agree.” After reverse coding appropriately, a mean item score is calculated, and higher scores reflect greater stigma; Cronbach’s alpha = 0.82.

*HIV testing.* Participants were asked how many times they had tested for HIV in the past 6 months. They were instructed not to include HIV testing that was conducted as part of the study procedures.

*Relationship characteristics*. Participants who reported being in a relationship were asked whether they were married to their partner, the length of the relationship, whether they live with their partner, and whether they have a sexual agreement with their partner that allows for sex with other people (classified as non-monogamous in a binary variable for analysis).

*Demographics.* We assessed age, race, ethnicity, formal education, current employment, state and city or town of residence (which was used to designate the national region of their residence and whether they lived in an urban or rural setting based on population size and density), and relationship status. Although we assessed ethnicity and race separately, for analysis we created a variable that combined the two so that we had a single categorical variable that represented men who identified as White (non-Hispanic), Black, Hispanic, Asian/Pacific Islander, or other mixed race/ethnicity that did not include any of the prior listed identities.

### Data Analysis

The main independent variable in the analysis was the categorization of IPV victimization and IPV perpetration. For both variables, participant self-reported presence of any victimization or perpetration at each of the three assessments was used to classify the participant as experiencing no IPV (no IPV at all three assessments), intermittent IPV (any IPV at one or two assessments, but not all three), or persistent IPV (any IPV at all three assessments). Descriptive statistics were used to characterize the sample with regard to IPV and the other constructs measured. One-way ANOVA was used to compare the three IPV groups (across the categorization of victimization and perpetration, in separate analyses) on continuous and binary measures, and Bonferroni tests were used to examine pairwise group comparisons when the F tests of the ANOVA were significant at *p *< .05.

Separate mixed effect models were conducted to examine the independent predictive value of IPV persistence categorization (separately for victimization and perpetration) on the dependent variables (current PrEP use, perceived partner support for PrEP use, PrEP stigma, and number of HIV tests in the past 6 months), while accounting for repeated measures among each person (random effects) (Schober & Vetter, 2018). Main effects were measured for time, intermittent IPV, and persistent IPV (no IPV was the reference). To explore sexual agreement for non-monogamy as a moderator of the IPV effect on the dependent variables, an indicator for the presence of a non-monogamy agreement at baseline and its interaction with both intermittent and persistent IPV were added to the model. All models included the baseline measures of age, education (binary indicator of at least 4 years of college), employment, minority race/ethnicity (binary indicator of non-Hispanic White or not), and relationship length (categorized as < 1 year, 1–6 years, and 7 or more years) as covariates; the models examining HIV testing also included current PrEP use at baseline as a covariate, because HIV testing is often a requirement for continued PrEP prescriptions.

Fit statistics for each model were inspected. For the model with the number of HIV tests in the past 6 months as the dependent variable, two outliers were identified and were truncated to the next highest value in the data. Otherwise, each model met its respective fit criteria (i.e., no overdispersion or zero inflation for Poisson models, homoscedasticity and normal errors for linear models, and linearity of log-odds for logistic models). Analyses were done using RStudio version 2024.04.2 + 764.

## Results

### Sample Characteristics

A total of 500 men enrolled in the study, of whom 448 (89.6%) completed assessments at each of the three time points (baseline, month 6, and month 12). Of these 448, 272 (60.7%) reported being in a relationship with the same partner at each assessment; one of these participants did not provide IPV data and was excluded from the analysis, resulting in a final analytic sample of 271 participants. Table 1 shows the baseline characteristics of the analytic sample, including sociodemographics, relationship characteristics, and the dependent variables in our analysis. The sample has representation across the four regions of the USA: 31.0% in the South, 28.4% in the West, 25.5% in the Northeast, and 15.1% in the Midwest. Mean age was 33.2 years (SD = 6.0), 81.6% had completed at least four years of college, 87.1% were salaried or self-employed, 91.1% self-identified as gay, and nearly a third (30.3%) were in their current relationship for over seven years. Racial and ethnic identification was diverse, with two-thirds (63.5%) being racial or ethnic minorities, including 32.1% Hispanic, 12.9% Black, and 14.8% Asian or Pacific Islander.

**Table 1:** Baseline characteristics of the sexual minority men who were in the same relationship throughout the 12-month study period (*n *= 271)

Characteristic	Mean (SD)/*n* (%)
Age (in years)	33.2 (6.0)
*Race/ethnicity*	
Non-Hispanic White	99 (36.5%)
Black/African-American	35 (12.9%)
Hispanic/Latino	87 (32.1%)
Asian/Pacific Islander	40 (14.8%)
Multiracial/Other	10 (3.7%)
At least 4 years of college	221 (81.6%)
Employed or self-employed	236 (87.1%)
Self-identified as gay	247 (91.1%)
*Region of residence*	
Northeast	69 (25.5%)
South	84 (31.0%)
West	77 (28.4%)
Midwest	41 (15.1%)
Reside in urban location^a^	58 (21.5%)
Currently married^b^	87 (32.1%)
*Length of relationship*^b^	
< 1 year	33 (12.3%)
1–6 years	154 (57.2%)
7 + years	82 (30.3%)
Cohabitates with partner^c^	196 (73.4%)
Sexual agreement with partner for non-monogamy^d^	151 (56.3%)
*Use of PrEP:*^a^	
Ever on PrEP	171 (63.3%)
Currently on PrEP	110 (40.7%)
PrEP Stigma (range: 1–5)	2.02 (0.56)
*Among those not on PrEP (n* = *160):*	
Partner disapproves of PrEP use	9 (5.6%)
Partner support for PrEP use (range: 1–5)	4.04 (0.81)
Tested for HIV in past 6 months^a^	184 (68.4%)

^a^*n *= 270 (1 missing); ^b^*n *= 269 (2 missing); ^c^*n *= 267 (4 missing); ^d^*n *= 268 (3 missing)

### Persistence of Intimate Partner Violence Victimization and Perpetration

Any IPV victimization was reported by 83 (30.6%) at baseline, 72 (26.6%) at month 6, and 69 (25.5%) at month 12. Examining the presence of any IPV victimization across each of the three assessments, 149 (55.0%) reported no IPV victimization, 82 (30.3%) reported intermittent IPV victimization (i.e., at one or two of the assessments), and 40 (14.8%) reported persistent IPV victimization (i.e., at each of the three assessments). Any IPV perpetration was reported by 60 (22.1%) at baseline, 52 (19.2%) at month 6, and 51 (18.8%) at month 12; 178 (65.7%) reported no IPV perpetration across all assessments, 73 (26.9%) reported intermittent IPV perpetration, and 20 (7.4%) reported persistent IPV perpetration. About two-thirds (62.3%) of those reporting either intermittent or persistent victimization also reported intermittent or persistent perpetration of IPV, although only one-third (32.5%) of those who reported persistent victimization also reported persistent perpetration.

### Intimate Partner Violence Persistence and its Association with PrEP Measures and HIV Testing

*PrEP use.* At baseline, 171 (63.3%) reported ever using PrEP, including 110 (40.7%) who were currently using PrEP. PrEP stigma was significantly higher among those not on PrEP at baseline (M [SD] = 2.17 [0.57]), compared to those on PrEP (M [SD] = 1.79 [0.46]; *t *= 6.0 [d*f *= 259.3]; *p *< .001]). Table 2 lists rates of PrEP use across the categories of IPV persistence for victimization and perpetration at each assessment. At month 6, PrEP use varied significantly across the categories of both IPV victimization (*F* = 3.1 [2, 266]; *p* = .048) and IPV perpetration (*F* = 4.2 [2, 266]; *p *= .02); however, pairwise comparisons showed only a significant difference with greater PrEP in the intermittent IPV perpetration subgroup compared to no IPV perpetration (*p *= .01).

**Table 2:** Bivariate analysis of the categorization of persistent intimate partner violence victimization and perpetration, and associations with HIV prevention behaviors and attitudes

	IPV victimization				IPV perpetration			
	No IPV (*n *= 149)	Intermittent IPV (*n *= 82)	Persistent IPV (*n *= 40)	*F*(df); *p*	No IPV (*n *= 178)	Intermittent IPV (*n *= 73)	Persistent IPV (*n *= 20)	*F*(df); *p*
*On PrEP:*								
Baseline	40%	43%	40%	0.09 (2, 267); .91	39%	48%	30%	1.4 (2, 267); .26
M6	37%	51%	54%	3.1 (2, 266); .048	38%	58%	47%	4.2 (2, 266); .02
M12	43%	54%	53%	1.6 (2, 267); .21	45%	55%	50%	1.1 (2, 267); .34
*If not on PrEP: Partner support for PrEP use:*								
Baseline	4.2 (0.8)	4.1 (0.8)	3.6 (0.9)	2.3 (2, 58); .11	4.2 (0.7)	3.6 (1.0)	3.7 (0.9)	4.2 (2, 58); .02
M6	4.3 (0.7)	3.6 (0.9)	3.6 (1.0)	3.4 (2, 54); .01	4.2 (0.8)	3.8 (0.9)	3.0 (0.6)	4.0 (2, 54); .03
M12	4.1 (0.8)	3.7 (0.9)	3.3 (0.6)	2.9 (2, 43); .06	4.1 (0.8)	3.5 (1.1)	3.2 (0.6)	2.7 (2, 43); .08
*PrEP stigma*								
Baseline	1.90 (.51)	2.06 (.52)	2.37 (.66)	12.7 (2, 266); < .001	1.9 (0.5)	2.1 (0.6)	2.3 (0.6)	6.1 (2, 266); .002
M6	–	–	–	–	–	–	–	–
M12	1.91 (.56)	2.06 (.51)	2.32 (.65)	8.8 (2, 264); < .001	2.0 (0.6)	2.1 (0.5)	2.2 (0.6)	1.6 (2, 264); .20
*HIV tests in past 6 months*								
Baseline	1.3 (1.1)	1.1 (1.0)	1.1 (0.9)	0.6 (2, 266); .55	1.2 (1.0)	1.2 (1.0)	1.2 (0.9)	0.1 (2, 266); .91
M6	1.2 (1.3)	1.1 (0.9)	1.5 (1.9)	1.2 (2, 267); .31	1.2 (1.2)	1.4 (1.6)	1.4 (1.0)	0.6 (2, 267); .55
M12	1.3 (1.2)	1.2 (1.0)	2.0 (3.7)	3.3 (2, 267); .04	1.2 (1.1)	1.7 (2.9)	1.7 (1.2)	2.2 (2, 267); .12

*IPV* Intimate partner violence; *PrEP* Pre-exposure prophylaxis; *M6* Month 6; *M12* Month 12

In the multivariate models without the moderator, time was the sole significant predictor of PrEP use across the 12-month observation period, with PrEP use increasing over time in both the IPV victimization (Adj. OR [95% CI] = 1.69 [1.21–2.36]; see Table 3] and IPV perpetration (Adj. OR [95% CI] = 1.69 [1.21–2.36]; see Table 4) analyses. When sexual agreement for non-monogamy was added to the models as a moderator, it was a significant predictor of PrEP use in both the IPV victimization (Adj. OR [95% CI] = 44.7 [7.86–253.8]) and IPV perpetration (Adj. OR [95% CI] = 58.0 [11.67–288.0]) analyses, but its interactions with intermittent and persistent IPV were not associated with PrEP use; time remained an independent predictor of PrEP use. In all models, the main effects of intermittent and persistent IPV were not associated with PrEP use.

**Table 3:** Persistence of intimate partner violence victimization and its association with HIV prevention behaviors and attitudes, moderated by sexual agreement for non-monogamy

	Currently on PrEP		Partner supportive of PrEP use^a^		PrEP stigma		HIV tests in past 6 months	
	No moderator	With moderator	No moderator	With moderator	No moderator	With moderator	No moderator	With moderator
	OR (95% CI)	OR (95% CI)	Beta (SE); *p*	Beta (SE); *p*	Beta (SE); *p*	Beta (SE); *p*	Beta (SE); *p*	Beta (SE);* p*
Intercept	0.02 (0.00–1.51)	0.01 (0.00–0.35)	3.61 (0.52); < .001	3.82 (0.45); < .001	1.99 (0.20); < .001	2.05 (0.20); < .001	− 0.10 (0.28); .71	− 0.17 (0.27); .54
Time	1.69 (1.21–2.36)	1.70 (1.22–2.38)	− 0.11 (0.05); .02	− 0.09 (0.05); .04	0.00 (0.01); .95	0.001 (0.01); .94	0.05 (0.04); .16	0.05 (0.04); .16
Intermittent IPV victimization	2.28 (0.49–10.68)	0.85 (0.07–11.0)	− 0.33 (0.22); .13	− 0.48 (0.26); .07	0.13 (0.07); .054	0.30 (0.11); .01	− 0.08 (0.09); .40	− 0.05 (0.18); .80
Persistent IPV victimization	1.92 (0.27–13.76)	1.39 (0.08–23.9)	− 0.77 (0.25); .004	− 0.64 (0.30); .04	0.43 (0.09); < .001	0.50 (0.14); .004	0.15 (0.12); .20	0.45 (0.19); .01
Non-monogamy	–	44.7 (7.86–253.8)	–	0.73 (0.22); .001	–	− 0.17 (0.08); .04	–	0.33 (0.12); .007
Intermittent IPV-V * Non-monogamy	–	1.18 (0.05–26.50)	–	0.04 (0.38); .92	–	− 0.19 (0.14); .18	–	− 0.12 (0.21); .55
Persistent IPV-V * Non-monogamy	–	0.80 (0.02–31.30)	–	− 0.32 (0.43); .46	–	− 0.08 (0.18); .64	–	− 0.52 (0.23); .03
On PrEP at baseline	–	–	–	–−	–	–	0.92 (0.08); < .001	0.83 (0.09); < .001

^a^Among participants not on PrEP

*IPV* Intimate partner violence; *PrEP* Pre-exposure prophylaxis; *OR* Odds ratio; *CI* Confidence interval; *SE* Standard error; *IPV-V* IPV victimization

All models included the following covariates: age, race, relationship duration, employment status

**Table 4:** Persistence of intimate partner violence perpetration and its association with HIV prevention behaviors and attitudes, moderated by sexual agreement for non-monogamy

	Currently on PrEP		Partner supportive of PrEP use^a^		PrEP stigma		HIV tests in past 6 months	
	No moderator	With moderator	No moderator	With moderator	No moderator	With moderator	No moderator	With moderator
	OR (95% CI)	OR (95% CI)	Beta (SE); *p*	Beta (SE); *p*	Beta (SE); *p*	Beta (SE); p	Beta (SE); *p*	Beta (SE); *p*
Intercept	0.02 (0.00–1.40)	0.00 (0.00–0.27)	3.59 (0.51); < .001	3.62 (0.43); < .001	2.01 (0.21); < .001	2.07 (0.21); < .001	− 0.15 (0.28); .60	− 0.18 (0.28); .52
Time	1.69 (1.21–2.36)	1.70 (1.22–2.38)	− 0.12 (0.05); .02	− 0.09 (0.05); .06	0.00 (0.01); .94	0.00 (0.01); .92	0.05 (0.04); .16	0.05 (0.04); .16
Intermittent IPV perpetration	2.99 (0.64–14.06)	2.36 (0.20–27.89)	− 0.55 (0.23); .02	− 0.93 (0.27); .001	0.12 (0.07); .09	0.03 (0.12); .79	0.09 (0.09); .35	0.42 (0.16); .01
Persistent IPV perpetration	0.87 (0.06–11.86)	3.68 (0.13–103.48)	− 0.64 (0.31); .045	− 0.11 (0.41); .79	0.31 (0.12); .01	0.34 (0.17); .04	0.33 (0.16); .04	0.46 (0.22); .04
Non-monogamy	–	58.0 (11.67–288.0)	–	0.57 (0.18); .003	–	− 0.24 (0.08); .002	–	0.35 (0.12); .003
Intermittent IPV-P * Non-monogamy	–	0.61 (0.03–12.75)	–	0.79 (0.38); .04	–	0.18 (0.15); .24	–	− 0.51 (0.20); .01
Persistent IPV-P * Non-monogamy	–	0.07 (0.00–6.78)	–	− 1.03 (0.53); .06	–	− 0.06 (0.24); .79	–	− 0.24 (0.31); .43
On PrEP at baseline	–	−	–	−	–	–	0.92 (0.09); < .001	0.84 (0.09); < .001

^a^Among participants not on PrEP

*IPV* Intimate partner violence; *PrEP* Pre-exposure prophylaxis; *OR* Odds ratio; *CI* Confidence interval; *SE* Standard error; *IPV-P* IPV perpetration

All models included the following covariates: age, race, relationship duration, employment status

*Perceived partner support for PrEP use. *Among participants who were not on PrEP, perceived partner support for the respondent starting PrEP varied significantly across the categorization of persistent IPV victimization at month 6 (*F* = 3.4 [2, 54]; *p* = .01), and across the categorization of persistent IPV perpetration at baseline (*F* = 4.2 [2, 58]; *p* = .02) and month 6 (*F* = 4.0 [2, 54]; *p* = .03) (see Table 2). At those assessments, participants reporting intermittent or persistent IPV reported less partner support for PrEP compared to those reporting no IPV (*p* values < .05).

In the multivariate models without the moderator, for both the analyses of IPV victimization (see Table 3) and IPV perpetration (see Table 4), time (victimization: beta [SE] =  − 0.11 [0.05]; *p* = .02; perpetration: beta [SE] = -0.12 [0.05]; *p* = .02) and persistent IPV (victimization: beta [SE] =  − 0.77 [0.25]; *p* = .004; perpetration: beta [SE] =  − 0.64 [0.31]; *p* = .045) were predictive of lower partner support; intermittent IPV was also predictive of lower partner support in the IPV perpetration analysis (beta [SE] =  − 0.55 [0.23]; *p* = .02). When sexual agreement for non-monogamy was added to the models as a moderator, it predicted greater partner support in both the IPV victimization (beta [SE] = 0.73 [0.22]; *p* = .001] and IPV perpetration (beta [SE] = 0.57 [0.18]; *p* = .003) analyses; its interaction with intermittent IPV was also significantly associated with partner support in the IPV perpetration analysis (beta [SE] = 0.79 [0.38]; *p *= .04), such that the presence of intermittent IPV perpetration was associated with greater partner support for PrEP when a sexual agreement for non-monogamy was present.

*PrEP stigma. *PrEP stigma differed significantly across the categorization of persistent IPV victimization at baseline (*F* = 12.7 [2, 266]; *p *< .001) and month 12 (*F* = 8.8 [2, 264]; *p *< .001), and across the categorization of persistent IPV perpetration at baseline (*F* = 6.1 [2, 266]; *p *= .002) (see Table 2). At each of these assessments, the persistent IPV group had significantly higher levels of stigma than the no IPV group in both the victimization (*p *< .001) and perpetration (*p *= .01) analyses; the persistent IPV victimization group also had higher stigma than the intermittent group (*p *< .001).

In multivariate analysis, models that excluded the moderator showed that persistent IPV was positively associated with greater stigma in both the IPV victimization (beta [SE] = 0.43 [0.09]; *p *< .001; see Table 3) and IPV perpetration (beta [SE] = 0.31 [0.12]; *p *= .01; see Table 4) analyses. When sexual agreement for non-monogamy was added to the models as a moderator, it was an independent predictor of lower stigma in both the IPV victimization (beta [SE] =  − 0.17 [0.08]; *p *= .04) and IPV perpetration (beta [SE] = −0.24 [0.08]; *p *= .002) analyses, but its interactions with intermittent and persistent IPV were not associated with PrEP stigma. Persistent IPV remained a significant predictor in both the IPV victimization (beta [SE] = 0.50 [0.14]; *p* = .004) and IPV perpetration (beta [SE] = 0.34 [0.17]; *p* = .04) analyses; intermittent IPV victimization was also predictive of greater stigma (beta [SE] = 0.30 [0.11]; *p *= .01).

*HIV testing*. At baseline, over two-thirds of the sample (*n *= 184; 68.4%) reported testing for HIV in the past 6 months. In bivariate analysis, the only significant group difference was the number of HIV tests in the 6 months prior to month 12 (*F* = 3.3 [2, 267]; *p *= .04]), which varied significantly across the categorization of IPV victimization (see Table 2); those experiencing persistent IPV reported significantly more HIV testing compared to the intermittent group (*p *= .048).

In multivariate analysis, models that excluded the moderator showed that the use of PrEP was a significant predictor of more HIV testing in both the IPV victimization (beta [SE] = 0.92 [0.08]; *p *< .001; see Table 3) and IPV perpetration (beta [SE] = 0.92 [0.09]; *p *< .001; see Table 4) analyses. The only other significant predictor was persistent IPV perpetration, which was also associated with more HIV testing (beta [SE] = 0.33 [0.16]; *p *= .04). When sexual agreement for non-monogamy was added to the models as a moderator, it was an independent predictor of more HIV testing in both the IPV victimization (beta [SE] = 0.33 [0.12]; *p *= .01) and IPV perpetration (beta [SE] = 0.35 [0.12]; *p *= .003) analyses; its interactions with persistent IPV victimization (beta [SE] =  − 0.52 [0.23]; *p *= .03) and intermittent IPV perpetration (beta [SE] = − 0.51 [0.20]; *p *= .01) were also significant predictors, such that the presence of such IPV levels was associated with less HIV testing when a sexual agreement for non-monogamy was present.

## Discussion

This is one of the first longitudinal studies of IPV among HIV-negative SMM in relationships, and its association with utilization and attitudes related to tools for HIV prevention. Level of IPV persistence was unrelated to PrEP use; however, persistent IPV victimization and perpetration were both associated with lower perceived partner support for PrEP use among those not using PrEP, as well as greater PrEP stigma. Conversely, persistent IPV victimization and perpetration were associated with more frequent HIV testing. Together, these findings suggest a complex and nuanced influence of IPV on the use of HIV prevention tools. The presence of a sexual agreement for non-monogamy, which was a strong predictor of more HIV testing and PrEP use, acted as a moderator of the relationships between IPV and HIV testing and partner support for PrEP, albeit in mixed ways, highlighting the critical role it may have for HIV prevention among coupled SMM.

Consistent with other studies of SMM (Liu et al., 2021; Walters et al., 2013), one-quarter to one-third of the sample reported IPV victimization, and one-fifth reported IPV perpetration, at each assessment in the study, but following these men over 12 months enabled us to examine the persistence of IPV. IPV victimization was persistent (i.e., present at each of the three assessments over 12 months) in one-third of those experiencing any victimization, while IPV perpetration was persistent in one-quarter of those reporting any perpetration. These findings suggest that among SMM, most of whom had been with their partners for several years, both victimization and perpetration are typically not persistent and stable, but intermittent and months can pass between events of violence.

In contrast to other studies of SMM (Hong et al., 2022, 2023), including those in relationships which found rates of PrEP use ~ 15–20% (Starks et al., 2019), much higher utilization was reported in this study. Nearly half of the men reporting PrEP use at each of the assessments and use increased over the course of the study. Furthermore, two-thirds of the sample reported any lifetime use of PrEP. Some studies have shown that single SMM are more likely to use PrEP (Hong, 2024), which further accentuates our finding among coupled men. One possible reason for this finding is that over half of our sample reported being in a relationship that had a sexual agreement for non-monogamy—a factor that has been shown to be predictive of PrEP use, in this study and others (Kahle et al., 2020).

Both IPV victimization and perpetration were not significantly associated with PrEP use over time in this study, but persistent experience with both forms of IPV was associated with factors that may influence PrEP use. PrEP stigma was greater among men who reported persistent IPV victimization, as well as those who reported persistent IPV perpetration. Furthermore, both persistent victimization and perpetration were associated with perceived lower partner support for PrEP use by the respondent, among PrEP non-users. Both PrEP stigma (Pico-Espinosa et al., 2022) and low partner support for PrEP (Kahle et al., 2020) have been found to impede PrEP use among SMM; therefore, these findings highlight how persistent IPV may indirectly pose a barrier to PrEP use.

Although prior cross-sectional studies have documented inconsistent associations between IPV and PrEP use (Hong, 2024; Storholm et al., 2025), our longitudinal study provides new evidence that the influence of IPV on PrEP may be less about its direct effect on PrEP utilization and more about the interpersonal and psychological factors that shape its use; such factors may include stigma, trust, and power dynamics that influence open communication or lack thereof. IPV may undermine PrEP uptake, not only through reduced access or awareness, but by eroding partner support and amplifying perceived stigma, both of which are key determinants of PrEP decision making in couples. Dyadic models of HIV prevention among SMM promote open communication and shared decision making as key pathways influencing prevention behavior (Darbes et al., 2014; Starks et al., 2019), which may be impeded by IPV and its related control and power dynamics (Bosco et al., 2022; Goldenberg et al., 2016; Jessup et al., 2024).

PrEP use was a strong predictor of more frequent HIV testing, which is not surprising given that HIV testing is often a requirement to maintain receipt of PrEP prescriptions. Controlling for PrEP use, both persistent IPV victimization and persistent IPV perpetration were associated with more HIV testing, suggesting a recognition of greater vulnerability or risk for HIV acquisition in the context of a tumultuous or violent relationship. However, among participants who had a sexual agreement for non-monogamy, persistent IPV victimization and intermittent IPV perpetration were each associated with less HIV testing, despite the increased HIV risk posed by sex partners outside the relationship. This pattern contrasts with research suggesting that SMM in consensual non-monogamous relationships typically test more frequently (Kahle et al., 2020), and may indicate that IPV-related stress, control or conflict in the relationship could interfere with health-seeking behaviors even when HIV risk is high. In other words, violence within non-monogamous partnerships may undermine the communication and trust needed to sustain routine testing practices. Further research is needed to better understand the interplay between IPV and use of HIV prevention strategies in the context of non-monogamy.

Among coupled SMM, sexual agreements for non-monogamy have been shown to be significantly associated with a greater likelihood of PrEP use (Kahle et al., 2020), and this was true in this study as well. Agreement for non-monogamy was not only predictive of PrEP use, but also lower PrEP stigma, greater perceived partner support for PrEP, and more frequent HIV testing. Sexual partners from outside of the relationship may heighten the risk for HIV and other sexually transmitted diseases; therefore, greater use and support for HIV prevention tools such as HIV testing and PrEP may represent a rational strategy for maintaining sexual health and reducing anxiety related to HIV risk in couples that have such an agreement (Guo et al., 2022; Starks et al., 2019; Stephenson et al., 2015, 2022). Conversely, without sexual agreements that allow outside partners, PrEP use may be perceived as a sign of infidelity or mistrust (Kahle et al., 2020; Storholm et al., 2023). Like the finding related to HIV testing, sexual agreement for non-monogamy also moderated the relationship between IPV and perceived partner support for PrEP, as the presence of such an agreement reversed the direction of this relationship—intermittent perpetration and persistent victimization each became associated with greater perceived partner support for PrEP. The increased HIV risk presented by non-monogamy, together with the volatility associated with IPV, may influence partners to more favorably view the benefits of PrEP.

Taken together, these findings illustrate the bidirectional and context-dependent associations between IPV and HIV prevention engagement among coupled SMM. IPV can simultaneously heighten risk awareness (increasing HIV testing) and erode trust and open communication (reducing PrEP support). This underscores the importance of relationship context, power dynamics, and communication in shaping prevention behavior decision making and the need for interventions that address relationship function alongside biomedical HIV prevention.

Programs and healthcare providers looking to promote PrEP and HIV testing among coupled SMM should incorporate screening for both IPV victimization and perpetration, particularly when men express stigmatized attitudes toward PrEP use or perceived low partner support for PrEP. In fact, HIV testing presents a salient opportunity for IPV screening. Furthermore, decisions for PrEP use among men in relationships are inherently going to be influenced by relationship factors, including partner attitudes toward PrEP, such as perceived benefits as well as possible threats regarding relationship trust. Promoting open candid communication in the relationship, and establishment of sexual agreements that meet the desires and intentions of both partners, should be encouraged to help couples adopt sexual health and HIV prevention behaviors such as PrEP use and periodic HIV testing. Moreover, long-acting formulations of PrEP may be introduced as an option for SMM who are high in PrEP stigma or perceived low partner support for PrEP. While sexual agreements are common among SMM couples, HIV risk reduction strategies are not always discussed or included in such agreements (Rios-Spicer et al., 2019). This highlights both the value of such discussions and open communication for HIV prevention (Mitchell et al., 2016), and the potential route by which IPV and its impedance of open communication may act as a barrier to HIV prevention.

From a clinical and public health standpoint, these results reinforce the need for trauma-informed, couple-centered HIV prevention strategies. Clinicians providing PrEP and HIV testing services to SMM should be trained to screen for IPV in ways that are sensitive to sexual and gender minority identities and that integrate discussions of safety, communication, and relationship dynamics into counseling sessions. Providers can also normalize conversation about sexual agreements and PrEP use within relationships to reduce stigma and foster mutual support. Integrating IPV screening and prevention into HIV testing and sexual health settings can help to identify men whose IPV experiences may be barriers to sustained HIV prevention engagement. At a systems level, partnership between IPV services, LGBTQ+ community organizations, and HIV prevention programs can help to ensure coordinated and affirming care for SMM. This includes training staff on IPV assessment and intervention for clients in same-gender relationships, ensuring privacy and safety protocols, and linking clients with affirming mental health and trauma recovery services.

Several study limitations are worth considering. While the analysis focused on relationship and partner factors influencing the use of HIV prevention behaviors, only one member of the dyad was included in the study. Partner inclusion in the assessments may have bolstered the validity of partner-related measures; however, their inclusion in research pertaining to IPV can pose unacceptable safety risks. The overall sample was large and diverse in many ways, but the sample was not diverse on some factors that affected our statistical power as well as generalizability: Some of the IPV subgroups (e.g., persistent perpetration) were small, which limited the statistical power to assess the associations in the analysis; most of the men were recruited from sexual networking platforms, which may have skewed the sample toward coupled men who are non-monogamous; and most of the men were well educated and self-identified as gay, which limited our ability to examine how low socioeconomic status and other sexual identities may moderate the association between IPV and uptake of HIV prevention tools. We did not assess whether perpetration of violence was a response to the need for self-defense, which would enable us to better classify such behaviors—particularly among those who also reported physical forms of victimization. The self-report measure of IPV is limited by its susceptibility to social desirability bias and underreporting of IPV victimization and perpetration, and its lack of assessment of severity and frequency. Other measurement constraints included the absence of measures of relationship trust, communication, and power related to decision making, which could inform the associations observed in this study.

In conclusion, this longitudinal study of IPV among coupled SMM found that intermittent or persistent IPV victimization and perpetration were not directly associated with PrEP use over the 12-month study period. However, persistence of both forms of IPV was related to lower perceived partner support for PrEP use and greater PrEP stigma, thereby indirectly impeding PrEP use. Furthermore, persistent IPV victimization and perpetration were each associated with more frequent HIV testing. Sexual agreement for non-monogamy was a strong predictor of more HIV testing and PrEP use, and acted as a moderator of the relationships between IPV and HIV testing and partner support for PrEP, albeit in mixed ways as it presented a context for greater perceived partner support for PrEP, but less frequent HIV testing. These findings highlight the importance of addressing interpersonal violence, relationship power and communication, and stigma reduction as integral components of HIV prevention for SMM couples. IPV screening, relationship counseling, and trauma-informed strategies need to be integrated into biomedical HIV prevention interventions (including long-acting injectable PrEP), to ensure that advances in PrEP delivery reach those most affected by violence and HIV.

## Data Availability

De-identified dataset and statistical code are available to researchers upon submission of proposal and review by the study team.
